# In vitro efficacy of cetyltrimethylammonium bromide (CETAB)-modified nano-montmorillonite against aflatoxin B1 associated toxicity and methanogenesis

**DOI:** 10.1186/s12917-025-04546-w

**Published:** 2025-03-08

**Authors:** Yosra A. Soltan, Amr S. Morsy, Nesrein M. Hashem, Mahmoud A. I. Elazab, Mohamed A. Sultan, Amr El-Nile, Gomaa Abo El Lail, Nagwa El-Desoky, Nourhan S. Hosny, Ahmed M. Mahdy, Elsayed E. Hafez, Sobhy M. A. Sallam

**Affiliations:** 1 Animal and Fish Production Department, Faculty of Agriculture, Alexandria University, Alexandria, Egypt; 2 Livestock Research Department, Arid Lands Cultivation Research Institute, City of Scientific Research and Technological Applications, Alexandria, Egypt; 3 Economic and Agribusiness Department, Faculty of Agriculture, Alexandria University, Alexandria, Egypt; 4 Soil and Water Sciences Department, Faculty of Agriculture, Alexandria University, Alexandria, Egypt; 5 Plant Protection and Biomolecular Diagnosis Department, Arid Lands Cultivation Research Institute, City of Scientific Research and Technological Applications, Alexandria, Egypt

**Keywords:** Clays, Nano-particles, Quaternary ammonium salt, Methane, AFB1

## Abstract

**Background:**

Modified nano-montmorillonite is gaining attention as a feed additive for its benefits on ruminal fermentation. Chemical and mechanical methods were used to modify montmorillonite. Cetyltrimethylammonium bromide (CETAB) was utilized for chemical modification, while grounding was carried out to achieve the desired nanoscale particle size, resulting in the formation of the nanoscale powder known as MNM_CETAB_. Impacts of MNM_CETAB_ supplementation on a basal diet, either contaminated with aflatoxin B1 (AFB1) or not at a level of 20 ppb were tested. Treatments included control (no supplements), a diet with 5 g per kilogram of dry matter (DM) of natural montmorillonite (NM), and diets with MNM_CETAB_ at two doses, 0.5 (low) and 1 (high) grams per kilogram DM.

**Results:**

The MNM_CETAB_ showed better physicochemical traits than NM clay, including narrower particle size range, higher cation exchange capacity (CEC), greater specific surface area (SSA), and more functional groups. A significant linear decreasing effect (*P* < 0.05) of MNM_CETAB_ addition on methane (CH) production was observed by the increasing level of the MNM_CETAB_ clay. The control diet contaminated with AFB1 resulted in lower fiber degradability than the other treatments (*P* < 0.05). No variations were observed in ruminal protozoal counts by both clay supplementations, although there was a noticeable trend (*P* = 0.08) towards reduced protozoal populations due to AFB1 contamination. AFB1-contaminated diets showed indications of reduced (*P* < 0.05) levels of total volatile fatty acids (VFA), and concentrations of butyrate and propionate (*P* < 0.05), alongside shifts towards elevated (*P* = 0.006) acetate levels, while the low dose of MNM_CETAB_ exhibited higher (*P* < 0.01) propionate concentrations than the other treatments.

**Conclusion:**

These findings underscored the anti-methanogenic properties and the favorable impacts of MNM_CETAB_ in mitigating the adverse impacts of AFB1on ruminal fermentation and nutrient degradability.

## Background

As greenhouse gas emissions (GHG) continue to rise, the issue of mycotoxins becomes increasingly significant. Global warming could potentially lead to shifts in the geographical distribution of mycotoxigenic microorganisms. This poses a substantial peril to health and the economy, as well as a significant risk to the safety of global food supplies [1]. In the domain of livestock production, approximately 81% of GHG originate from the process of rumen microbial methanogenesis, resulting in the production of methane (CH_4_), a highly potent GHG [2, 3]. Furthermore, the production of CH_4_ in the rumen leads to an energy loss that could potentially have been harnessed for animal growth or milk production [3]. Aflatoxin B1 (AFB1) stands out as one of the most potent hepato-carcinogenic and immuno-suppressive compounds [4]. Moreover, the ability of ruminal microorganisms to degrade AFB1 is limited, of utmost significance is its capacity to undergo metabolism, giving rise to AFM1, a compound renowned for its established carcinogenic and mutagenic properties [5]. Feeding on diets contaminated with AFB1 often results in negative effects (e.g., reduced ruminal fiber degradability, inhibition in fermentation patterns, decreased feed intake, and animal productivity) [2, 5]. Consequently, it appears that strategies aimed at mitigating both ruminal GHG and mycotoxins are increasingly pertinent for the foreseeable future [2]. Therefore, various feed additives have been utilized to address this issue. Furthermore, with the recent advancements in nanotechnology, the application of nanomaterials has shown promise in enhancing the effectiveness of feed additives targeted at modulating rumen fermentation pathways to optimize animal productivity. Notably, clay minerals have emerged as significant components within this context [6–8].

Clays (e.g., Montmorillonite) are widely considered safe for consumption by humans as well as animals [7–9]. Among these, Montmorillonite is widely available globally and is affordable [7, 9]. Numerous investigations have confirmed the impacts of NM on reducing AFB1 contamination [6, 7]. However, their influence on ruminal fermentation or methanogenesis appears to be limited. Noteworthy, Tate et al. [10] pioneered the utilization of NM to reduce emissions of ruminal CH_4_. Their findings suggest that NM exhibited the least effectiveness in suppressing methanogenesis among various clay types, likely attributed to its lower antimicrobial activity. However, its exceptional capacity for organic modifications distinguishes it, allowing effective alteration through ion exchange reactions with either anionic or cationic surfactants [8]. Considering that the most potent CH_4_ inhibitors typically comprise compounds featuring quaternary ammonium or nitrile (C ≡ N) functional groups, known for their direct CH inhibition mechanisms [6, 8], it is observed that they operate either as hydrogen-consuming agents or disruptors of the cell membranes on Gram-positive bacteria [6]. Thus, the modification of NM by incorporating these functional groups moieties using cationic surfactants, such as quaternary ammonium salts like CETAB. can augment its antimicrobial activity and CH_4_ inhibitory properties. Montmorillonite modified possesses higher cation exchange capacity (CEC), an affinity for aflatoxin contaminants, and antimicrobial efficiency against Gram-positive bacteria when compared to the natural state [8, 9, 11]. Furthermore, the mechanical reduction of clays into nano form has been scientifically established to enhance their physicochemical, adsorption, and anti-methanogenic properties [2, 8, 12]. Our previous research has demonstrated the improved ruminal fermentation pattern and nutrient degradability, and CH_4_ reduction through the utilization of modified nano-montmorillonite with CETAB (MNM_CETAB_). The higher negative charge and increased presence of hydroxyl groups in MNM_CETAB_ can enhance capacity to bind acidic H+, thereby favoring microbial fiber degradation while mitigating methanogenesis compared to NM [6, 8]. Nonetheless, the efficacy of MNM_CETAB_ in diets contaminated with AFB1 has not been empirically tested to date, to the best of our current knowledge. Therefore, it was hypothesized that MNM_CETAB_ as a feed additive might decrease the harmful impacts of AFB1 on ruminal fermentation and degrdability while inhibiting CH_4_ production. This study aims to elucidate the effects of modified clays on ruminal fermentation and nutrient degradation in diets contaminated with AFB1.

## Methods

This research took place at the Nanotechnology and Greenhouse Gases Laboratory, Faculty of Agriculture, Alexandria University, Egypt.

### Development of the MNMCETAB feed additive

The natural unmodified Ca-montmorillonite (NM, 95% purity) was sourced commercially from Egypt Bentonite and Derivatives Co. Inc., located in Alexandria, Egypt. The product of MNM_CETAB_ was developed using CETAB that obtained from Sigma Aldrich Co., Irvine, UK [8]. 10 g of NM was mixed in 600 milliliters of distilled water for 24 h at ambient temperature with a magnetic stirrer (Model 508, Globe Scientific Inc., New Jersey, USA). The required quantity of CETAB was slowly incorporated into the mixture and stirred for 5 h at 80 °C, followed by 12 h at room temperature using the same magnetic stirrer. The resulting material was subsequently filtered, washed, and dried at 90 °C for 24 h. To obtain the final product MNM_CETAB_ product, the dried clay underwent wet grinding with isopropanol utilizing a high-energy planetary ball mill (Photon Ball Mill Model PH-BML 912, Photon Scientific Co., Egypt) operating at a rated speed of 300 ± 10% revolutions per minute and a jar rotation speed of 450 ± 10% rotations per minute. The milling process was conducted for a total duration of 400 min, with alternating cycles of 5 min of milling followed by 10 min of rest, utilizing a 100 ml capacity zirconia ball milling jar and zirconia balls.

### Characterization of clay feed additives

The particle size distribution (D) and SSA of the clay products were analyzed with a laser particle analyzer (Bettersize 2600; Dandong Bettersize Scientific Ltd., Dandong, China). The values of pH of the experimental NM and MNM clays measured using a CRISON GLP 21 model pH meter from Barcelona, Spain. The CEC of the clays was analyzed using Rhoades’ method [13]. The surface charge was assessed using Zeta potential analysis conducted with a Malvern ZETASIZER Nano series instrument from Worcestershire, UK [8].

To identify the elemental contents of the experimental clays, both NM and MNM_CETAB_ clay samples were determined using an energy-dispersive X-ray (EDX) system coupled with a scanning electron microscope (SEM; Jeol JSM-6360 LA, Tokyo, Japan). The nanoparticle morphology of the clay samples was examined using SEM analysis. Before imaging, the clay samples were coated with gold to enhance resolution, following the procedure outlined by Soltan et al. [2]. Fourier Transform Infrared Spectroscopy (FTIR) was used to identify the functional groups in the clays. An infrared spectrometer (Shimadzu FTIR-8400 S, Osaka, Japan) equipped with a deuterated triglycine sulfate detector and purge gas generator was employed.

### The experimental treatments

For the in vitro assay, a basal diet was formulated according to the nutrient requirements of lactating sheep, following guidelines established by the National Research Council (NRC) [14]. It consisted of 350 g of berseem (*Trifolium alexandrinum*) clover hay, 150 g of wheat straw, 225 g ground maize, 165 g wheat bran, 90 g of soybean meal, 10 g of calcium carbonate, and 10 g of mineral premix with vitamins per kilogram DM. The diet was measured according to AOAC [15] and contained the following per kilogram of DM, 924 g of organic matter (OM), 138 g of crude protein (CP), 425 g of neutral detergent fiber (NDF), 214 g of acid detergent fiber (ADF), 49.8 g of acid detergent lignin (ADL), and 35 g of ether extract (EE). The NDF, ADF, and ADL were sequentially determined exclusive of residual ash using ANKOM filter bags [16]. The total aflatoxin content of the diet was 13.9 ppb, which was extracted using VICAM immunoaffinity columns (VICAM Aflatest, Milford, USA) following the VICAM fluorometry method [17].

The effects of supplementing clay feed additives on the basal diet prepared with or without contamination, with a final concentration of AFB1 set at 20 ppb, were evaluated in eight treatments: control basal diet (zero supplementation), NM supplemented at 5 g/kg DM basal diet, and MNM_CETAB_ supplemented at low level and high level (0.5 and 1 g/kg DM basal diet), respectively. The final total aflatoxins concentration in the basal diet contaminated with AFB1 was 33.9 ppb, exceeding the maximum allowable levels for total aflatoxins (20 ppb) and AFB1 (10 ppb) in dairy animal diets as per Egyptian regulations [18]. The experimental supplementation level of NM was determined [7], while the MNM_CETAB_ clay levels were analyzed according to Soltan et al. [2]. The AFB1 used in the experiment (98% purity) was derived from *Aspergillus flavus* (Sigma Chemical Co, St. Louis, Missouri, USA).

### Gas production procedure

To evaluate the experimental treatments, the semi-automatic gas production (GP) system was employed, following the method outlined by Bueno et al. [19] and customized for this study as per Soltan et al. [20]. The experiment of GP was carried out as a single run, with all experimental treatments completed within one day. To ensure consistent rumen environmental conditions, ruminal contents were collected individually from three buffalo calves (460 ± 6 SE kg) that were fasted and subsequently slaughtered at the experimental station’s slaughterhouse of the Alexandria University. These calves had been fed *ad libitum* with a local commercial diet for beef production. The ruminal contents were immediately transferred to thermo-containers (39 °C), flushed with CO_2_, and transported to the laboratory. The ruminal contents from the these calves were mixed in equal parts (1:1:1) for 10 s and then filtered through three layers of cheesecloth. The resulting inoculum was kept in a water bath maintained at 39 °C with continuous CO_2_ flushing. For each experimental treatment, twelve glass incubation bottles (120 ml volume each) were prepared. An amount of 0.5 g of each treatment was weighed and subsequently transferred into the incubation bottle. Following this, 15 mL of rumen inoculum and 30 mL of Menke’s buffer solution were added to each bottle, creating a 75 ml headspace [20]. The bottles were securely sealed with aluminum seals and 20 mm butyl rubber stoppers. The incubation was perfromed for 24 h (39 °C) in a forced air incubator (OCT 100, Chem-Tech Inc., Kafr El Sheikh, Egypt). Similar procedures were applied to the blank bottles, which contained only buffer solution and rumen inoculum, and the internal standard bottles, containing buffer solution, rumen inoculum, and Egyptian berseem clover hay. Blank bottles were utilized to determine net gas production values, while internal standard bottles were employed to correct for sensitivity differences caused by the inocula.

### Gas and CH4 production determination

The gas pressure was measured at 3, 6, 9, 12, and 24 h after the beginning of incubation by a pressure logger (model PSI-V2, SmartLab, Cairo, Egypt). The gas volume was calculated using the following equation: GP (ml) = 6.5465 × *p* − 0.9573 (*n* = 600; R^2^ = 0.99), where GP represented the gas volume in milliliters, and p was the measured pressure in psi.

For CH_4_ determination, one milliliter of the headspace gas was collected at each pressure measuring time using a 3 ml syringe and transferred to 5 ml vacutainer tubes. Concentrations of CH_4_ were measured using gas chromatography (GC) analysis performed with an Agilent Greenhouse Gas Analyzer, supplied by Agilent Technologies, Inc. (Santa Clara, California, USA).The details of the GC separation conditions used in this study have been described in a previous publication [2].

### Rueminal degradability, fermentation characteristics, and count of protozoa

To inhibit the ruminal microbial activity, all incubation bottles were promptly placed on ice after the 24 h incubation period. To evaluate nutrient degradability, the contents of the incubation bottles underwent treatment with a neutral detergent solution at 90 °C for 3 h [21]. The undegraded residues were collected from the bottles in pre-weighed crucibles, washed sequentially with hot distilled water and acetone, and dried at 70 °C for 48 h. Following this, the residues were incinerated at 600 °C for 2 h. The quantities of true degraded organic matter (TDOM) and true degraded neutral detergent fiber (TDNDF) were determined by subtracting the amounts of non-degraded organic matter and non-degraded neutral detergent fiber from the amounts initially incubated, as previously described [20].

The pH values were measured using a the same pH meter utilized for determining the pH of the clay products. Ammonia concentrations were measured using a commercial kit from Biodiagnostic Inc., Giza, Egypt. The VFA values were analyzed using gas chromatography (GC; Scion 456-GC/FID, Netherlands) [22]. The GC was outfitted with a capillary Rt-2560 column (100 m × 0.25 mm ID, 0.20 μm df, Restek) and operated with a consistent helium flow of 1.2 ml/min as the carrier gas. The total protozoal count was determined through microscopy [23].

### Statistical analysis

All data were analyzed using the MIXED procedure of SAS (SAS Institute Inc., Cary, USA, version 9.0) with one-way ANOVA. The incubation bottle was considered as the experimental unit. The data were analyzed as a completely randomized design with repeated measures by the following model: Yijkl = µ + Di + Tj + Iik + (D × T)ij + eijkl, where Yijkl represents the observation; µ is the overall mean; Di is the fixed effect of the treatment; Tj is the fixed effect of AFB1 supplementation; Iik is the random effect of the treatment; (D×T) ij is the interaction effect between treatmentand AFB1 supplementation, and eijkl is the residual error. Orthogonal contrast statements were developed to evaluate the linear and quadratic responses of each dependent parameter to incremental levels of MNM_CETAB_. Statistical significances were defined as *P* ≤ 0.05, with trends noted at *P* ≤ 0.10.

## Results

### Characterization of NM and MNMCETAB

**Fig. 1:**
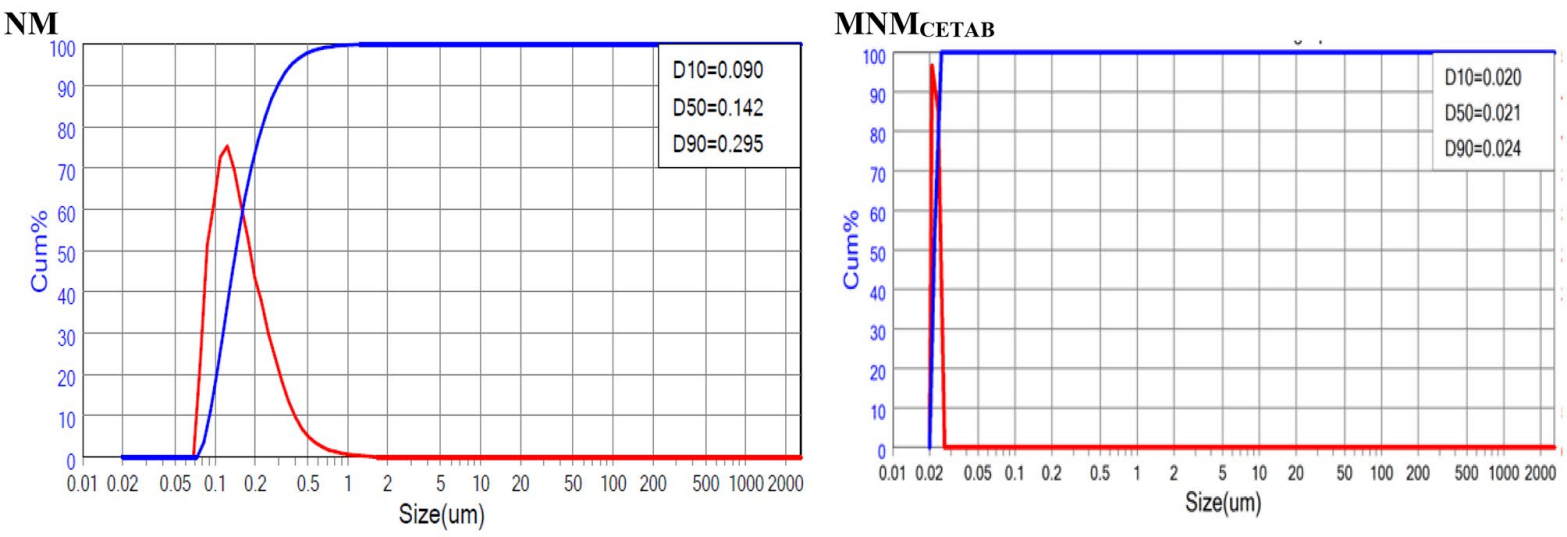
Particle size distribution graph for the natural montmorillonite (NM) and modified nano-montmorillonite by cetyltrimethyl ammonium bromide (MNM_*CETAB*_)

**Fig. 2:**
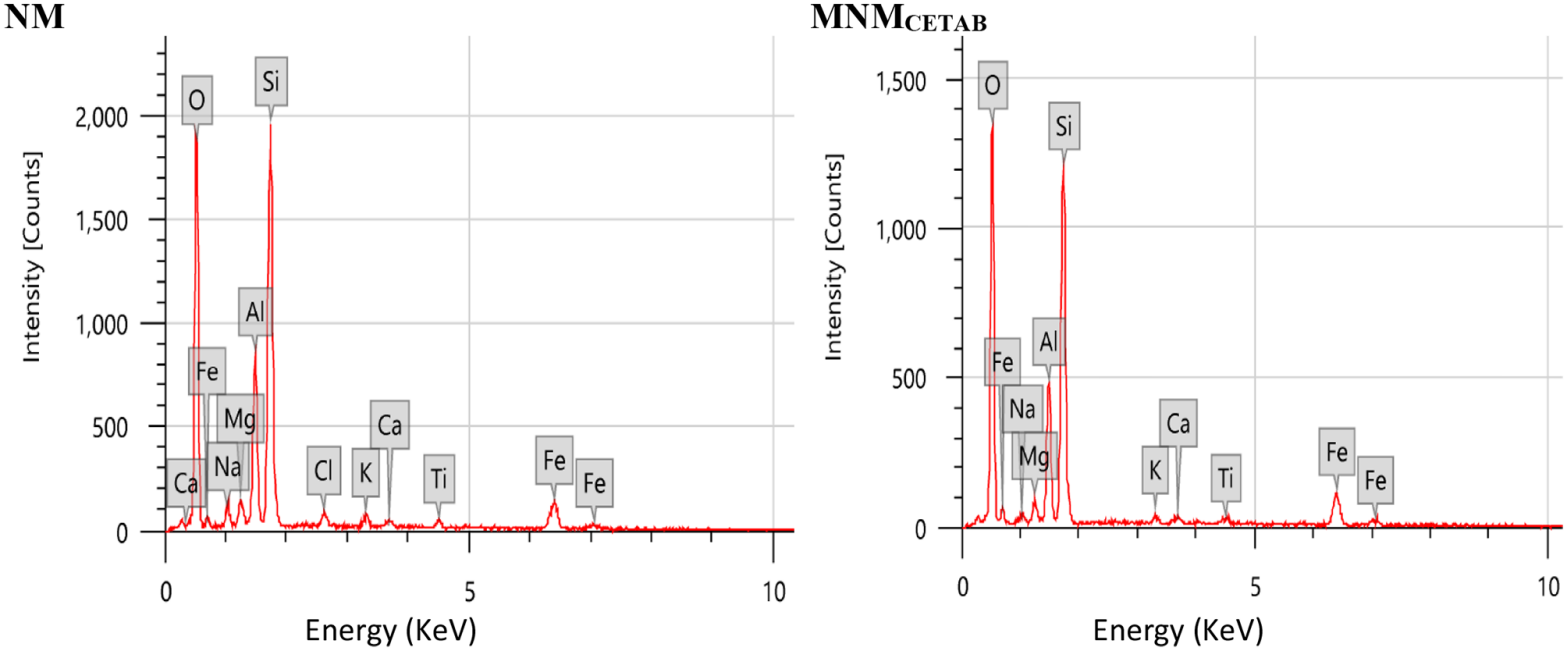
Energy dispersive x-ray (EDX) for the natural montmorillonite (NM) and modified nano-montmorillonite by cetyltrimethyl ammonium bromide (MNM_*CETAB*_)

Table 1 presents the physicochemical characteristics of the clay products. Figure 1 illustrates the size distribution., which indicated a broad distribution in case of NM clay while the distribution became narrower for MNM_CETAB_. The particle size at D90 decreased from 295 nm in NM to 24 nm in MNM_CETAB_. It is worth noting that the modifications made to Montmorillonite in the current study had a significant effect on its specific surface area, as evidenced by the higher specific surface area of MNM_CETAB_ compared to NM. There were minimal pH changes observed among all clay products. However, MNM_CETAB_ showed a higher CEC than NM (82.3 vs. 77.4 meq/100 g, respectively). The Zeta potential of NM clay was negative, and this negativity increased further following modification with CETAB. Figure 2 shows the EDX survey scan results for the experimental clays, with the corresponding elemental concentrations listed in Table 2. Chlorine (Cl) was only detected in NM and not in MNM_CETAB_, while the concentrations of oxygen (O), potassium (K), and iron (Fe) were found to be higher in MNM_CETAB_ than in NM.Conversely, the concentrations of silicon (Si), sodium (Na), calcium (Ca), aluminum (Al), and titanium (Ti) were lower in MNM_CETAB_ than in NM.

**Table 1:** The particle size distribution and specific surface area for the natural montmorillonite (NM) and modified nano-montmorillonite by cetyltrimethylammonium bromide (MNM_CETAB_)

Items	Montmorillonite products	
	NM	MNM_CETAB_
Particle size distribution		
D10 (nm)	90	**20**
D50 (nm)	**142**	**21**
D90 (nm)	**295**	**24**
Specific surface area (m^2^/g)	**5.70**	**99.68**
pH	8.05	7.90
Cation exchange capacity (meq/100 g)	77.4	**82.3**
Zeta potential	-23.1	-27.0

**Table 2:** Elemental compositions of the natural montmorillonite (NM) and modified nano-montmorillonite by cetyltrimethylammonium bromide (OMNM_CETAB_) detected by energy dispersive x-ray spectrum (EDX)

Item	Montmorillonite products	
	NM	MNM_CETAB_
Element (atomic %)*		
O -2	66.39 ± 0.59	68.00 ± 0.70
Na + 1	1.78 ± 0.09	1.20 ± 0.10
Mg + 2	1.31 ± 0.07	1.32 ± 0.08
Al + 3	7.49 ± 0.14	6.97 ± 0.16
Si + 4	18.07 ± 0.21	17.92 ± 0.25
Cl -1	0.71 ± 0.04	0
K + 1	0.70 ± 0.04	0.73 ± 0.05
Ca + 2	0.41 ± 0.03	0.31 ± 0.04
Ti + 3	0.49 ± 0.03	0.34 ± 0.04
Fe + 2	2.64 ± 0.08	3.22 ± 0.11

*The values are given as atomic percentage concentrations (atomic %) and have been normalized to 100%

**Fig. 3:**
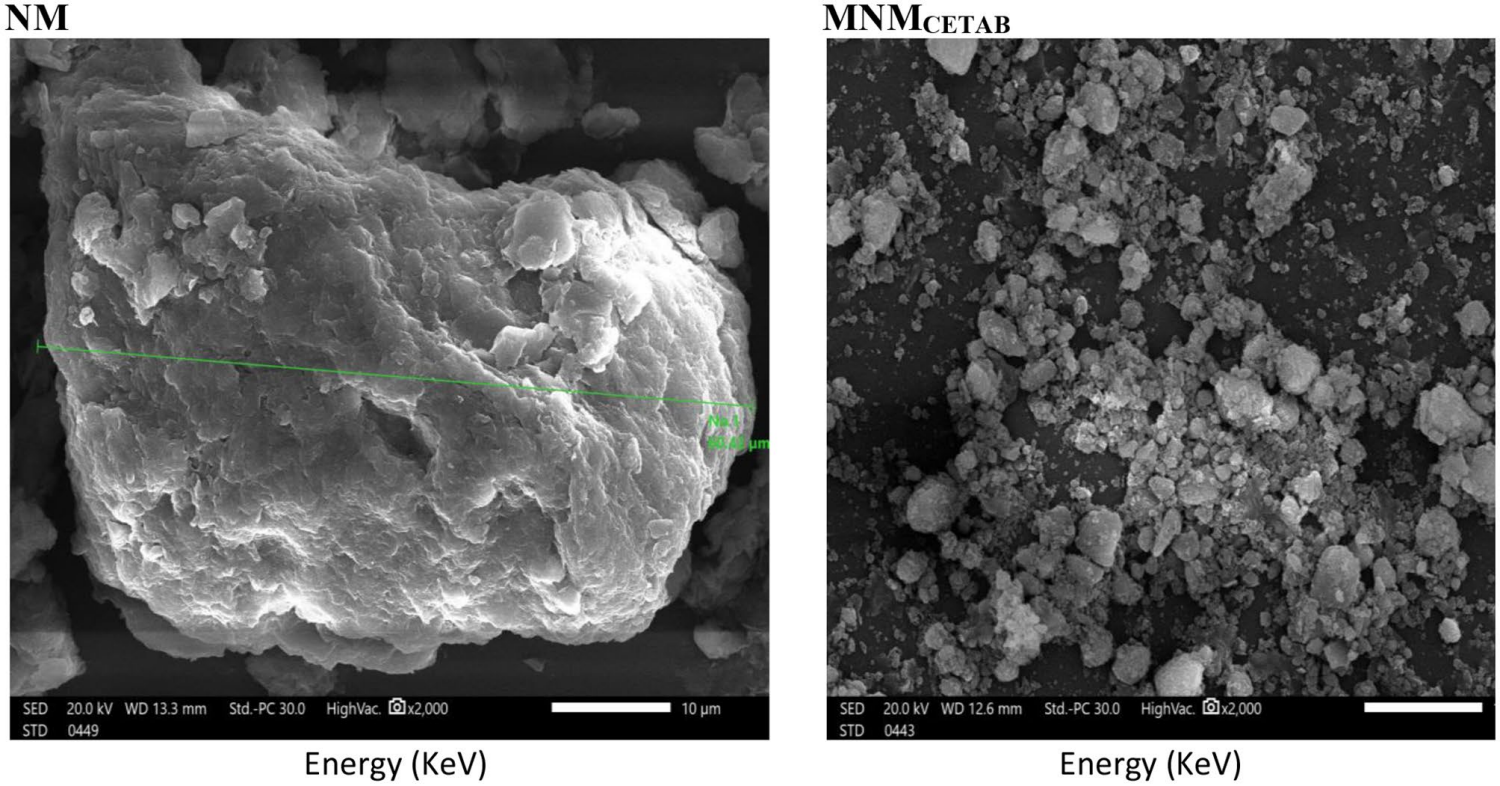
The surface morphology of the natural montmorillonite (NM) and modified nano-montmorillonite by cetyltrimethyl ammonium bromide (MNM_*CETAB*_)

Figure 3 displays SEM photomicrographs of the experimental clays, indicating significant alterations in the physical appearance of the clay particles post-modification with CETAB. Particle aggregates with larger particles and a relatively broader particle size distribution appeared in NM, meanwhile, the grain boundaries gradually disappeared, and the flakes of clay minerals dispersed throughout. MNM_CETAB_.

**Fig. 4:**
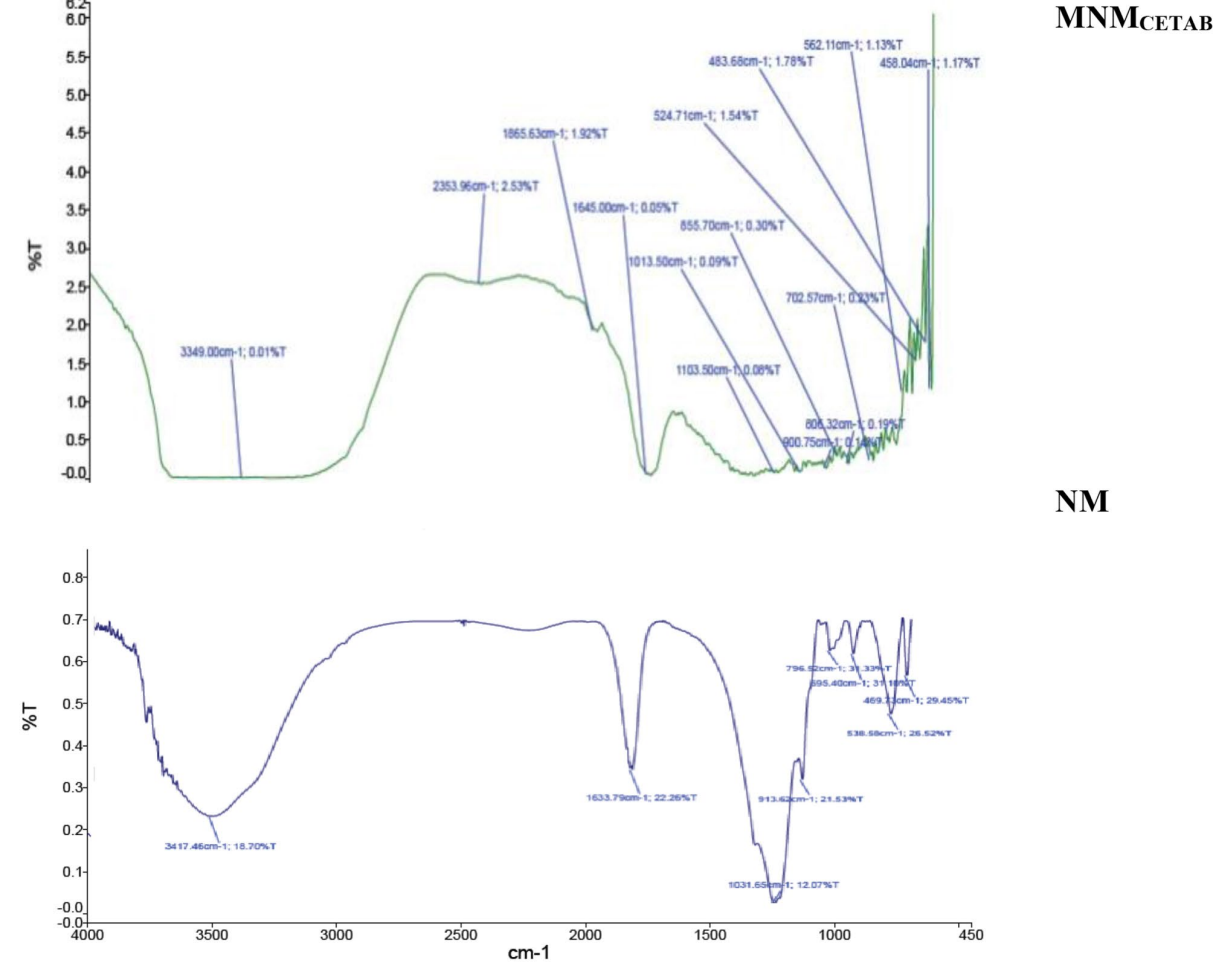
Fourier transform infrared spectroscopy (FTIR) analysis for the natural montmorillonite (NM) and modified nano-montmorillonite by cetyltrimethyl ammonium bromide (MNM_CETAB_)

Figure 4 displays the results obtained from the FTIR analysis of the NM and OMNM_CETAB_ clays. A total of 20 peaks within a frequency range of 454 to 3349 cm^− 1^ were detected in OMNM_CETAB_, whereas only 8 peaks within a frequency range of 469 to 3417 cm^− 1^ were detected in NM. In the high-frequency range, a well-defined peak related to the C ≡ N stretching functional group was solely observed in MNM_CETAB_ at 2353 cm^− 1^. Additionally, peaks associated with the C-N and C-Br stretching functional groups were detected in MNM_CETAB_ at (one peak), 1103 cm^− 1^(one peak), and 684 to 562 cm^− 1^ (5 peaks), whereas they were not detected in NM.

### Ruminal gas and methane production, and nutrient degradability

Table 3 results indicated that the impact of MNM_CETAB_ on GP and CH_4_ production differed from that of unmodified clay. The GP values were affected by the treatment, AFB1, and MNM_CETAB_dose × AFB1 interaction (*P* < 0.05), however, the contrast analysis did not reveal any significant effects. The diets contaminated with AFB1 significantly (*P* < 0.01) reduced GP values compared to uncontaminated diets. Treatment, MNM_CETAB_ dose, and MNM_CETAB_ dose × AFB1 interaction affected (*P* < 0.05) the CH_4_ production, while the addition of AFB1 had no significant effect. The low dose of MNM_CETAB_, whether with or without AFB1, and the high dose of MNM_CETAB_ with AFB1 addition, showed a similar significant reduction (*P* < 0.01) in CH_4_ production compared to the other treatments. Moreover, the contrast analysis revealed a linear reduction effect (*P* < 0.001) of MNM_CETAB_ addition on CH production. No significant effects of treatment, AFB1, MNM_CETAB_ dose, and MNM_CETAB_ dose × AFB1 interaction changes were noted on the values of TDOM. Similarly, the contrast analysis revealed that MNM_CETAB_ had no significant effect on TDOM. The control diet with AFB1 exhibited significantly reduce in TDNDF values (*P* = 0.01) compared to the NM diets, while neither AFB1 nor MNM_CETAB_ dose had any significant effect on TDNDF values. The contrast analysis indicated a tendency towards a quadratic increase (*P* = 0.08) in TDNDF values with the increasing dose of MNM_CETAB_.

**Table 3:** Supplementation effects of natural montmorillonite (NM) and modified nano-montmorillonite by cetyltrimethylammonium bromide (MNM_CETAB_*)* on ruminal gas production (GP), methane production (CH_4_); true degraded organic matter (TDOM) and true degraded neutral detergent fiber (DNDF)

Item	Treatments (T)									*P* value					
	Control		NM		MNM_CETAB_				SEM	T	MNM_CETAB_				
					Low		High				**Dose**	**AFB1**	**Dose×AFB1**	Contrast	
	**-AFB1**	**+AFB1**	**-AFB1**	**+AFB1**	**-AFB1**	**+AFB1**	**-AFB1**	**+AFB1**						**Linear**	**Quadratic**
GP (ml/ g IOM)	152	137	128	120	160	165	167	142	4.354	< 0.001	0.109	< 0.0001	< 0.0001	0.175	0.143
CH_4_ (ml/ g IOM)	16.7	17.2	16.7	16.7	14.3	14.88	15.03	12.3	0.478	< 0.001	< 0.0001	0.209	0.009	< 0.0001	0.113
Nutrient degradability															
TDOM	770	756	696	774	744	765	767	753	4.906	0.667	0.343	0.667	0. 100	0.60	0.1743
TDNDF	451	381	466	456	428	433	438	403	12.81	0.010	0.11	0.88	0.018	0.219	0.08

SEM = Standard error of the mean

Contrast: = effects of control (0 supplementation g/kg DM) compared with MNM_CETAB_ treatments

Low = MNM_CETAB_ supplemented at 0.5 g/ kg dry matter, High = MNM_CETAB_ supplemented at 1 g/ kg dry matter

### Ruminal fermentation and the count of protozoa

Table 4 presents the impacts of the experimental clay feed additives on ruminal ammonia, pH, count of protozoa, and VFA. Among the treatments, NM diets with or without AFB1 had the lowest (*P* < 0.01) ruminal pH values. The ammonia concentration was influenced by the treatment, MNM_CETAB_ dose, and MNM_CETAB_ dose × AFB1 interaction (*P* < 0.05); whereas, the addition of AFB1 did not have a significant effect. The contrast analysis indicated a trend towards a linear decrease (*P* = 0.09) and a quadratic decrease (*P* = 0.06) in ammonia concentration values as the MNM_CETAB_ dose increased. There were no differences found in the protozoal counts between the clay products either by clay or AFB1-supplemented diets. Among the different treatments, the AFB1-contaminated control had significantly lower VFA values (*P* < 0.01) compared to all clay-treated diets. The AFB1 contamination negatively affected the total VFA, although this effect was a tendency (*P* = 0.09). The concentrations of total VFA were influenced (*P* < 0.05) by the dose of MNM_CETAB_ and the interaction between MNM_CETAB_ dose and AFB1 contamination. Additionally, the contrast analysis indicated that there was a quadratic an increase in total VFA (*P* < 0.01) with increasing doses of MNM_CETAB_. Treatments with NM and the low dose of MNM_CETAB_, regardless of AFB1 presence, exhibited lower (*P* < 0.05) acetate concentrations than the control diet. AFBI resulted in increasing effects (*P* = 0.006) on acetate concentrations. There was an enhancement in the concentrations of acetate was observed with increasing doses of MNM_CETAB_, and this effect was found to be quadratic (*P* < 0.01). The low dose of MNM_CETAB_ had higher (*P* < 0.01) values of propionate than the other treatments. The presence of AFB1 in the contaminated diets led to a decrease (*P* = 0.02) in the concentrations of butyrate compared to the diets not contaminated with AFB1. A low dose of MNM_CETAB_ without AFB1 supplementation led to significantly (*P* < 0.05) lower levels of valeric acid and branched-chain fatty acids (BCFA; isobutyric and isovaleric) compared to the NM diet. The presence of AFB1 contamination further reduced the levels of BCFA (*P* < 0.05). However, contrast analysis indicated that the application of MNM_CETAB_ resulted in both a linear and quadratic increases (*P* < 0.05) in the levels of BCFA;.

**Table 4:** Supplementation effects of natural montmorillonite (NM) and modified nano-montmorillonite by cetyltrimethylammonium bromide (MNM_CETAB_*)* on ruminal pH, ammonia concentrations, total volatile fatty acids (VFA) concentration (mM), and molar proportions of individual VFA (% of total VFA)

Item	Treatments (T)									*P* value					
	Control		NM		MNM_CETAB_				SEM	T	MNM_CETAB_				
					Low		High				Dose	AFB1	Dose× AFB1	Contrast	
	**-AFB1**	**+AFB1**	**-AFB1**	**+AFB1**	**-AFB1**	**+AFB1**	**-AFB1**	**+AFB1**						**Linear**	Quadratic
Ruminal pH	5.91	5.93	5.84	5.87	5.90	5.93	5.93	5.93	0.01	< 0.001	0.478	0.135	0.7245	0.391	0.392
Ammoina (mg/100 ml)	28.5	24.6	22.02	22.3	26.5	27.7	24.6	26.5	0.920	0.0003	0.05	0.21	0.0496	0.099	0.06
Protozoa (105/ml)	6.97	7.12	5.92	6.07	7.27	4.50	7.35	5.25	0.771	0.328	0.541	0.081	0.363	0.482	0.396
VFA															
Total (mM)	54.68	46.4	64.5	64.2	63.7	63.4	56.9	56.5	2.850	< 0.001	< 0.0001	0.091	0.015	0.269	< 0.0001
Acetate, % of total	59.7	60.2	58.7	59.1	59.1	59.2	59.5	60.5	0.08	< 0.001	< 0.0001	0.006	0.0241	0.8507	< 0.0001
Propionate, % of total	19.5	19.7	19.04	19.1	19.9	20.16	19.3	19.19	0.120	< 0.001	< 0.0001	0.556	0.171	0.001	0.0003
Butyrate, % of total	12.1	11.8	12.6	12.3	12.2	12.2	12.3	12.3	0.285	0.010	0.090	0.023	0.122	0.186	0.0734
Isobutyrate, % of total	1.89	1.69	1.99	1.78	2.08	2.07	1.90	1.912	0.030	< 0.001	< 0.0001	0.048	0.03	0.010	< 0.0001
Valerate, % of total	1.56	1.49	1.56	1.48	1.71	1.69	1.605	1.55	0.068	0.007	< 0.0001	0.0613	0.587	0.0727	< 0.0001
Isovalerate, % of total	2.16	2.13	2.22	2.18	2.48	2.42	2.289	2.113	0.039	< 0.001	< 0.0001	0.0015	0.044	0.055	< 0.0001

SEM = standard error of the mean

Contrast: = effects of control (0 supplementation g/kg DM) compared with MNM_CETAB_ treatments

Low = MNM_CETAB_ supplemented at 0.5 g/ kg dry matter, High = MNM_CETAB_ supplemented at 1 g/ kg dry matter

## Discussion

The physicochemical characteristics of the modified MNM_CETAB_ exhibited notable distinctions from those of the unmodified clay. The modifications employing CETAB led to a narrower size distribution, reduced particle size, and enhanced specific surface area. These variances can be ascribed to the effectiveness of the wet grinding method utilizing isopropanol, which underwent an extension from 6 to 8 h in the current study compared to our previous approach [8]. Consequently, we anticipated observing differences in the physicochemical properties of MNM_CETAB_ compared to those observed in our prior study.

Reducing the particle size of MNM_CETAB_ contributes to an increase in its SSA, enhancing its activity by providing more surface area for chemical reactions [13]. This is evident in the higher CEC of MNM_CETAB_ compared to NM, indicating a greater the incorporation of metal hydrolysates and ions into the clay interlayer space, thereby enhancing the activity of the modified clay [24]. Consistent findings regarding the high CEC of MNM_CETAB_ were also reported in our previous study [2]. The EDX analysis confirmed the successful modification of Montmorillonite clay by CETAB. The increases in the percent weight of O, K, and Fe, and the decreases in Na, Al, Si, Ca, and Ti found in MNM_CETAB_ compared to NM support this suggestion [9]. These results also indicate that MNM_CETAB_ may have increased antibacterial activity compared to NM. Recent research indicates that increased oxygen content in modified clays can heighten their antibacterial effectiveness by generating reactive oxygen species (ROS) in oxygen-rich environments, which are detrimental to bacterial cells [24]. ROS can inflict damage on bacterial cell membranes, induce oxidative stress, and disrupt bacterial metabolic pathways, ultimately leading to cell death [6]. Furthermore, higher oxygen content may stimulate the production of oxygen-dependent enzymes, such as cytochrome c oxidase, further amplifying the antibacterial efficacy of these agents [25]. Moreover, the nanostructure of MNM_CETAB_, detected through SEM analysis, suggests its successful production in a nano form. This nanostructure may facilitate easier penetration of bacterial cell walls compared to NM, potentially altering its internalization mechanism and enhancing its antimicrobial potency [9, 11].

The profile of FTIR analysis can confirm the successful modification process in producing MNM_CETAB_, the presence of a new peak of the C ≡ N stretching functional group and other peaks associated with the C-N and C-Br stretching functional groups in MNM_CETAB_ may confirm such a suggestion. The C-Br stretching function group detected in MNM_CETAB_ can affect its antibacterial activity, as the bromine atom in this bond can act as an electron-withdrawing group, which can increase the electrophilicity of adjacent carbon atoms and make the MNM_CETAB_ more reactive toward nucleophiles [25]. This can lead to the formation of reactive intermediates that can damage bacterial cells and disrupt their metabolic processes [24]. Moreover, it was reported that agents containing C-Br stretching function groups may exhibit high lipophilic properties, which can enhance their ability to penetrate bacterial cell membranes and reach intracellular targets [25].

Both MNM_CETAB_ and NM exhibited negative charges as indicated by the Zeta potential analysis, with MNM_CETAB_ demonstrating a higher negative charge compared to NM. The elevated negative charge of MNM_CETAB_ can be attributed partly to increases in SSA, CEC, and functional group numbers, along with differences in the percentage weights of microminerals detected in MNM_CETAB_ compared to NM. These physicochemical characteristics of MNM_CETAB_ may enhance its absorptive capacity relative to NM. Additionally, these findings suggest that MNM_CETAB_ and NM may have differential effects on ruminal microbial fermentation, prompting further investigation into their respective impacts on ruminal fermentation characteristics.

The in vitro experiment results showed that the AFB1-contaminated control diet had lower GP values compared to the AFB1-free control diet, indicating that AFB1 could adversely affect the rumen fermentation processes since the volume of the net GP is the average of the microbial activity [27, 28]. Previous literature has documented the detrimental effects of AFB1 on GP using various contaminated concentrations. Khodabandehloo et al. [29] reported that AFB1 concentrations at 5 and 10 µg/ml, AFB1 decreased total in vitro GP. Similarly in another study by Mojtahedi et al. [30] reported a reduction in total GP from 196 to 166 ml/g DM with AFB1 addition levels ranging from 0 to 900 ng/ml.

In our study, the adverse effects of AFB1 were mitigated by MNM_CETAB_ supplementation, resulting in higher GP in AFB1 diets supplemented with MNM_CETAB_ compared to NM diets. These results indicated the higher potential of MNM_CETAB_ to eliminate the toxicity of AFB1 than NM. The obtained physicochemical characteristics of MNM_CETAB_ such as increases in SSA, CEC, and number of the functional groups in addition to the differences in the percent weights of the microminerals detected in MNM_CETAB_ compared to NM may lead to enhancing its AFB1 absorptive capacity compared to the NM [31].

The presence of AFB1 contamination had no impact on CH_4_ production. However, the contrast analysis demonstrated a linear decline in CH_4_ production due to the introduction of MNM_CETAB_. Importantly, this reduction did not negatively affect the GP or degradability of OM and NDF. This suggests that the decrease in CH_4_ levels caused by OMNM_CETAB_ was not attributed to microbial fermentation inhibition, but could be attributed to a potential influence on the methanogenesis process. However, this potential has not yet been substantiated, the identification of unique functional groups in OMNM_CETAB_ may support this hypothesis. Components containing C ≡ N functional groups were found to have a direct inhibition effect on ruminal CH_4_, as they serve a dual purpose: inhibit the activity of Gram-positive bacteria by affecting their cellular membrane [9], and act as a hydrogen-consuming agent, effectively lowering CH_4_ production [28]. Furthermore, OMNM_CETAB_ may act as a non-protein nitrogen source, thereby enhancing nitrogen availability for rumen microflora., promoting both ruminal microbial protein synthesis and fermentation parameters [27]. Additionally, compounds with C-Br stretching functional groups exhibit notable antibacterial properties against various bacterial species under anaerobic conditions such as the rumen [25].

The literature has documented a symbiotic relationship between protozoa and methanogens, with protozoa contributing essential metabolites like H_2_ to methanogenesis [32]. However, in this study, the total count of protozoa remained unaffected by the clay treatments or AFB1. Consequently, it suggests that protozoa may not have influenced the observed CH_4_ reduction. It’s worth noting that the 24-hour in vitro assay period might have been insufficient to explore the potential effects of these additives on protozoal levels. The effects of the clay feed additives and AFB1 contamination on ruminal pH may be associated with ammonia production [2, 28]. The pH reductions caused by NM were accompanied by declines resulted in changes in ammonia values, with observed increases in pH in AFB1-contaminated diets coincided with rises in ammonia levels. Generally, the pH values observed for all treatments were within the normal range of rumen pH values [26]. Organic components that contain C ≡ N and C-N stretching functional groups can be reduced to nitrite and ammonia in the rumen [30], consequently increasing the ammonia concentration. These findings may explain the increases in ruminal pH by the OMNM_CETAB_ compared to NM diets.This highlights the inherent potential of OMNM_CETAB_ for utilization, particularly during periods of anticipated elevated ruminal acidity risk (e.g., parturition, early lactation,….).

The current study results may indicate that our OMNM_CETAB_ product can alter VFA fermentative profile differently in the presence of AFB1, and this phenomenon is dose-dependent. Diets contaminated with AFB1 showed tendencies towards lower total VFA, propionate, and butyrate concentrations, along with shifts in the concentrations of individual VFA towards higher acetate levels. In normal rumen conditions, methanogenesis is the primary H_2_ sink, with propionate formation considered the main alternative. Butyrate and acetate have lower affinity for H_2_ utilization compared to methanogens [32]. In the present research, the decline in CH_4_ production was linked to the elevated molar ratio of propionate induced by the lower OMNM_CETAB_ dosage. However, this correlation did not hold for the higher dosage. Also, the OMNM_CETAB_ low dosage resulted in increasing the BCFA compared to other treatments. Sufficient levels of BCFA are essential for the synthesis of microbial protein in the rumen. This protein serves as a premium protein source, which is later accessible for digestion and absorption in the animal’s lower digestive tract [26].

## Conclusion

The physicochemical properties of NM clay were significantly enhanced through modification by CETAB and nano-grinding. The experimental MNM_CETAB_ exhibited higher CEC values, increased SSA, a narrower size distribution, and more functional groups (including C ≡ N, C-N, and C-Br stretching) compared to NM. Contamination of AFB1 in ruminant diets led to decreased GP, individual and total VFA, and total protozoal numbers. Supplementation of OMNM_CETAB_ in ruminant diets shows the potential to mitigate the adverse effects of AFB1 contamination, surpassing natural clay effects. Supplementation of MNM_CETAB_ at 0.5 g/kg emerges as a promising additive for reducing CH_4_ emissions and improving rumen fermentation, fiber degradation, and VFA production. However, these in vitro results do not encompass the potential impacts of MNM_CETAB_ on AFB1 metabolism and absorption in the lower gut and bloodstream. Therefore, further in vivo experiments with various diets are essential for a comprehensive understanding of modified clays’ utilization.

## Data Availability

Datasets generated and/or analyzed during this study are included in this article version, and if required any further information related to the data involved in the manuscript can be obtained from the corresponding author upon reasonable request.
